# Tipiracil binds to uridine site and inhibits Nsp15 endoribonuclease NendoU from SARS-CoV-2

**DOI:** 10.1038/s42003-021-01735-9

**Published:** 2021-02-09

**Authors:** Youngchang Kim, Jacek Wower, Natalia Maltseva, Changsoo Chang, Robert Jedrzejczak, Mateusz Wilamowski, Soowon Kang, Vlad Nicolaescu, Glenn Randall, Karolina Michalska, Andrzej Joachimiak

**Affiliations:** 1 Center for Structural Genomics of Infectious Diseases, Consortium for Advanced Science and Engineering, University of Chicago, Chicago, IL 60667 USA; 2 Structural Biology Center, X-ray Science Division, Argonne National Laboratory, Argonne, IL 60439 USA; 3 Department of Animal Sciences, Auburn University, Auburn, AL 36849 USA; 4 Department of Biochemistry and Molecular Biology, University of Chicago, Chicago, IL 60367 USA; 5 Department of Microbiology, Ricketts Laboratory, University of Chicago, Chicago, IL 60367 USA

**Keywords:** Molecular biology, Structural biology

## Abstract

SARS-CoV-2 Nsp15 is a uridine-specific endoribonuclease with C-terminal catalytic domain belonging to the EndoU family that is highly conserved in coronaviruses. As endoribonuclease activity seems to be responsible for the interference with the innate immune response, Nsp15 emerges as an attractive target for therapeutic intervention. Here we report the first structures with bound nucleotides and show how the enzyme specifically recognizes uridine moiety. In addition to a uridine site we present evidence for a second base binding site that can accommodate any base. The structure with a transition state analog, uridine vanadate, confirms interactions key to catalytic mechanisms. In the presence of manganese ions, the enzyme cleaves unpaired RNAs. This acquired knowledge was instrumental in identifying Tipiracil, an FDA approved drug that is used in the treatment of colorectal cancer, as a potential anti-COVID-19 drug. Using crystallography, biochemical, and whole-cell assays, we demonstrate that Tipiracil inhibits SARS-CoV-2 Nsp15 by interacting with the uridine binding pocket in the enzyme’s active site. Our findings provide new insights for the development of uracil scaffold-based drugs.

## Abstract (web summary)

Youngchang Kim, Jacek Wower, and colleagues explore the sequence specificity, metal ion dependence and catalytic mechanism of the Nsp15 endoribonuclease NendoU from SARS-CoV-2. The authors also solve five new crystal structures of the enzyme in complex with 5’UMP, 3’UMP, 5’cGpU, uridine 2′,3′-vanadate (transition state analog) and Tipiracil (uracil mimic), and demonstrate that Tipiracil inhibits SARS-CoV-2 Nsp15 by interacting with the uridine binding pocket in the enzyme’s active site.

## Introduction

The current pandemic of COVID-19 is caused by Severe Acute Respiratory Syndrome Coronavirus 2 (SARS-CoV-2). At the time of writing, there is no vaccine or proven drug against SARS-CoV-2. Since February 2020, concerted efforts have focused on characterizing its biology and developing various detection and treatment options, ranging from vaccines through antibodies to antivirals.

As a typical member of the *Coronaviridae* family, it is spherical, enveloped, non-segmented, (+) sense RNA virus with a large ~30 kbs genome. These genomes are used as mRNA for translation of a replicase-transcriptase complex (RTC) constituents made of two large polyproteins, Pp1a and Pp1ab, and as a template for replication of its own copy^1^. These polypeptides are processed by two viral proteases: papain-like protease (PLpro, a domain within non-structural protein 3 (Nsp3)), and 3C-like protease (Nsp5 or 3CLpro or Mpro). For CoV-2 the cleavage yields 15 Nsps, (Nsp11 is just a 7-residues peptide) that assemble into a large membrane-bound RTC that exhibits multiple enzymatic and binding activities. In addition, several sub-genomic RNAs are generated from (-) sense RNA during virus proliferation, resulting in translation of 4 structural and 9-10 accessory proteins. Non-structural proteins are potential drug targets for therapies and, because of their essentiality, sequence, and function conservation, developed therapeutics might in principle inhibit all human coronaviruses.

Nsp15 is a uridine-specific endoribonuclease. Its catalytic C-terminal domain shows sequence similarity and functionality of the EndoU family enzymes. They are involved in RNA processing and broadly distributed in viruses, archaea, bacteria, plants, humans, and other animals. The viral EndoU subfamily has been named NendoU. The Nsp15 enzyme is active as a 234 kDa hexamer consisting of three dimers. It was reported that NendoU cleaves both single- and double-stranded RNA at uridine sites producing 2′,3′-cyclic phosphodiester and 5′-hydroxyl termini^2^. The 2′,3′-cyclic phosphodiester is then hydrolyzed to 3′-phosphomonoester. In coronaviruses, toroviruses, and arteriviruses, NendoU is mapping to Nsp15s and Nsp11s. Nsp15s are much more sequence conserved than Nsp11s. It was proposed that in coronaviruses Nsp15 affects viral replication by interfering with the host’s innate immune response^3,4^. To evade host pattern recognition receptor MDA5 responsible for activating the host defenses, the Nsp15 cleaves the 5′-polyuridine tracts in (-) sense viral RNAs, which are the product of polyA-templated RNA synthesis^5^. These polyU sequences correspond to MDA5-dependent pathogen-associated molecular patterns (PAMPs) and NendoU activity limits their accumulation. For SARS-CoV it was reported that Nsp15 cleaves highly conserved non-translated RNA on (+) sense strand showing that both RNA sequence and structure are important for cleavage^6,7^.

Recently we have determined the first two crystal structures of SARS-CoV-2 Nsp15 and demonstrated that it is similar to the SARS-CoV and MERS-CoV homologs^8^. We have also shown that purified recombinant SARS-CoV-2 Nsp15 can efficiently cleave synthetic oligonucleotide substrate containing single rU, 5′−6-FAM-dArUdAdA−6-TAMRA-3′ ^8^. Studies of NendoU subfamily members, Nsp15 and Nsp11, indicate that the enzymes vary in their requirement for metal ion, with Nsp15 variants displaying dependence on Mn^2+^. At the same time, the proteins are often compared to metal-independent eukaryotic RNase A, which has a completely different fold, but shares active site similarities and broad function. Specifically, it carries two catalytic histidine residues, His12 and His119, that correspond to indispensable His250 and His235 in SARS-CoV Nsp15. RNase A cleaves its substrates in a two-step reaction consisting of transphosphorylation that generates 2′,3′-cyclic phosphodiester followed by hydrolysis leading to 3′-phosphoryl terminus. The latter step has only been reported for SARS-CoV Nsp15^9^. Here, we have expanded Nsp15 research to explore endoribonuclease sequence specificity, metal ion dependence, and catalytic mechanism. In addition to biochemical data, we report five new structures of the enzyme in complex with 5′UMP, 3′UMP, 5′GpU, uridine 2′3′-vanadate (UV)—a transition state analog, and Tipiracil—a uracil derivative. This compound is approved by FDA as a combination drug that is used with trifluridine in the treatment of colorectal cancer^10^. Tipiracil inhibits the enzyme thymidine phosphorylase which metabolizes trifluridine. We discovered that Tipiracil inhibits Nsp15 endoribonuclease activity, albeit not adequately to be an effective cure, but this scaffold can serve as a template for compounds that may block virus proliferation.

## Results and discussion

### Biochemical assays

#### Nsp15 nuclease activity

**Fig. 1:**
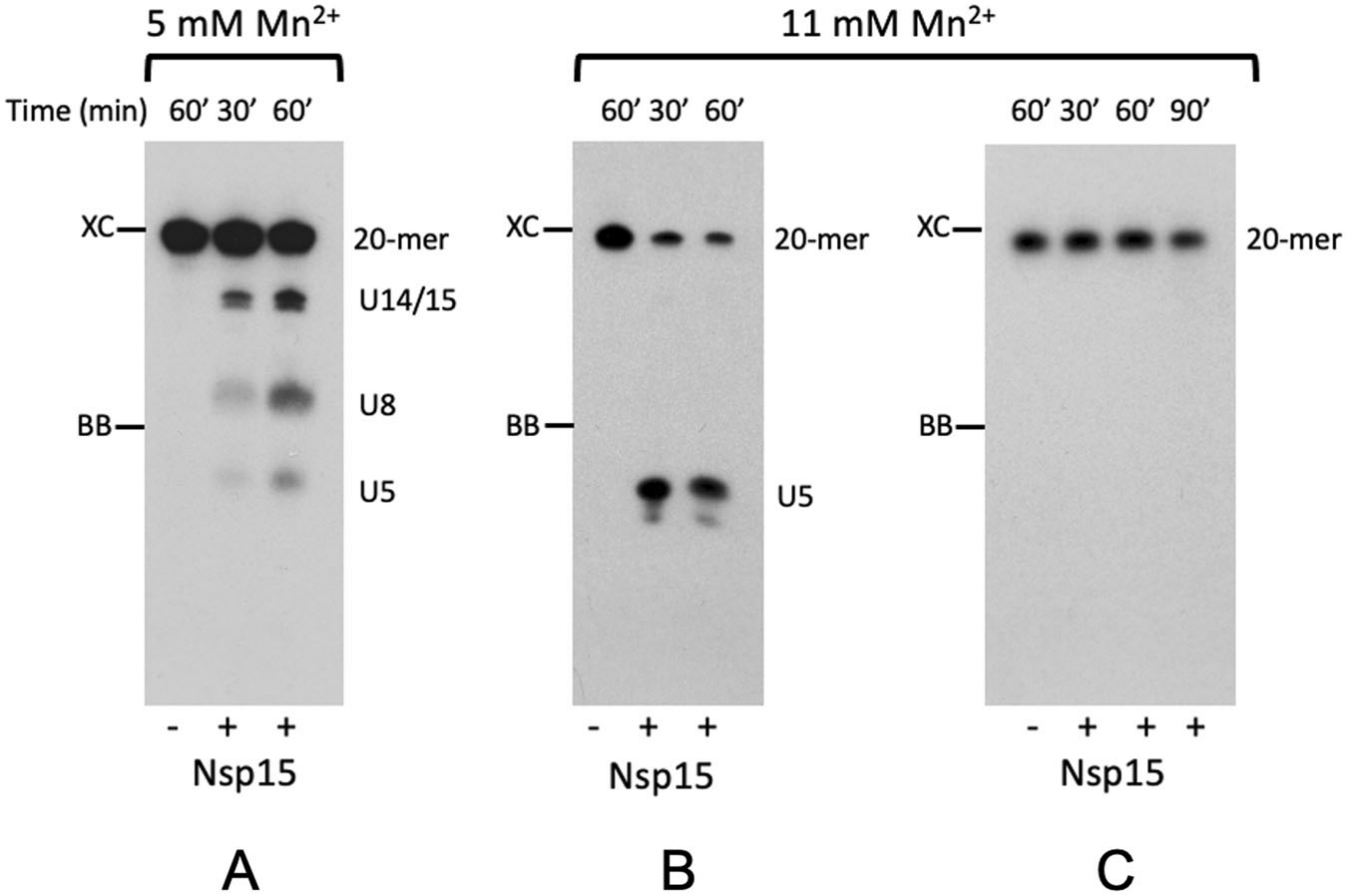
Uridine-specific endoribonuclease activity of SARS-CoV-2 Nsp15. 5′-^32^P-labeled RNA eicosamers were incubated at 37 °C with Nsp15. Reaction products were separated in an 20% polyacrylamide gel containing 7 M urea. Digestion products of (**A**, **B**) GAACU_5_CAUGG_10_ACCUU_15_GGCAG_20_ and (**C**) GAACA_5_CAAGG_10_ACCAA_15_GGCAG_20_, respectively. U5-, U8-, and U14/15- uridine-specific ladder. XC and BB mark the final positions of the xylene cyanole and bromophenol blue dyes. The cleavage of the eicosamer is uridine-specific and shows no sequence preference as 5′CU↓C, 5′AU↓G, 5’CU↓U, and 5′UU↓G are recognized and cut, especially at higher manganese concentration (compare Figs. 1A and 1B). Eicosamer that does not contain uridine is not cut (Fig. 1C).

We tested Nsp15 endoribonuclease activity using several assays and different substrates. We show that SARS-CoV-2 Nsp15 requires Mn^2+^ ions and retains only little activity in the presence of Mg^2+^ ions. The enzyme cleaves efficiently eicosamer 5′GAACU↓CAU↓GGACCU↓U↓GGCAG3’ at all four uridine sites (Fig. 1, Supplementary Figs. 1A and B), as well as synthetic EndoU substrate (5′−6-FAM-dArU↓dAdA−6-TAMRA-3′)^8^ in the presence of Mn^2+^, and the reaction rate increases upon rising metal ion concentration. The cleavage of the eicosamer seems to show no sequence preference as 5′CU↓C, 5′AU↓G, 5′CU↓U and 5′UU↓G are recognized and cut, especially at higher manganese concentration (Fig. 1). At 5 mM manganese, all four oligonucleotides accumulate, with the slowest cut sequence being the 5′AU↓G site. The eicosamer that does not have uridine in the sequence is not cleaved (Fig. 1, Supplementary Fig. 1C). The observed CoV-2 Nsp15 Mn^2+^ dependence is unlike its SARS-CoV homolog^9^. This is surprising as SARS-CoV-2 Nsp15 shares 88% sequence identity and 95% similarity with the SARS-CoV homolog and all active site residues are conserved. One explanation would be that the metal ion impacts the structure of RNA and the cleavage rate will depend on the sequence (and structure) of the substrate. Interestingly, by itself, the Nsp15 can cut RNAs at any uridine site, but as a component of RTC with Nsp8 and Nsp12, Nsp15 becomes a site-specific endonuclease which cuts RNA to leave short 5–10 uridine tails^5^. The enzyme also cuts the 3′ end of conserved non-coding region of RNA^6,7^ and the cleavage is RNA secondary structure-dependent.

Our observations provide new insights that might be relevant to Nsp15 roles in the SARS-CoV-2 reproduction cycle. First of all, earlier studies have demonstrated that coronavirus NendoU endonucleases are uridine-specific and display differences in their requirement for Mn^2+^ ions. On one side of the spectrum are Nsp11s of Equine Arteritis Virus (EAV), Porcine Reproductive and Respiratory Syndrome Virus (PRRSV), and Turkey Coronavirus Nsp15 that do not need Mn^2+^ for their endoribonuclease activity^11^. SARS-CoV-2 Nsp15 might represent the other extreme end of the range. Differences in the requirement for Mn^2+^ ions may affect the outcomes of the endoribonuclease activity. For example, metal-independent Nsp11 from EAV and some Nsp15s generate oligonucleotides with 2′,3′-cyclic phosphodiesters at their 3′ ends^9^. In contrast, metal-dependent SARS-CoV Nsp15 seems to be able to hydrolyze 2’,3’-cyclic phosphodiesters and produce oligonucleotides with 3’-phosphoryl groups on their 3′-ends^9^. By analogy, the SARS-CoV-2 homolog should follow a two-step reaction and our structures provide strong backing, but this hypothesis is yet to be tested as our assays do not discriminate between the two.

The requirement for Mn^2+^ ions by SARS-CoV-2 Nsp15 may by itself enhance pathogen infectivity because Mn^2+^ is required for the host innate immune responses to viral infections. RNA binding of SARS-CoV Nsp15 was reported to be enhanced in the presence of Mn^2+^, whereas RNA binding by the cellular XendoU homolog from the African clawed frog was not affected by Mn^2+^ ions^6,7^.

Nevertheless, the role of metal ion is puzzling as it was proposed that Nsp15 may have similar activity to eukaryotic RNase A. However, the RNase A activity is metal independent, perhaps suggesting that metal ions may help to position RNA for binding or cleavage. No metal ions or strong metal-binding sites were found in any of the models of EndoU family members available in the Protein Data Bank (PDB). Our efforts to locate Mn^2+^ ion in the Nsp15 structures also failed, arguing against a direct role of metal ions in catalysis. However, we observe the increase of Nsp15 thermal stability in the presence of such ions (Supplementary Fig. 3), though the molecular basis of this behavior remains enigmatic. Previous data for SARS-CoV Nsp15, Mn^2+^ ions were found to change the intrinsic tryptophan fluorescence of SARS-CoV Nsp15, indicating conformational changes^12,13^.

#### Inhibition by Tipiracil

**Fig. 2:**
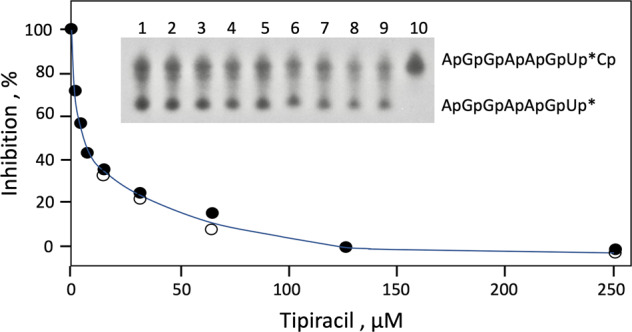
Inhibition of SARS-CoV-2 Nsp15 endoribonuclease by Tipiracil. RNA heptamers (5′AGGAAGU), labeled at their 3′ ends with 3′,5′-[5′-^32^P] bisphosphates to form octamers 5′ AGGAAGU^32^pCp, were incubated at 30 °C with Nsp15 in the presence of Tipiracil at 0, 1.85, 3.9, 7.8, 15.6, 31.25, 62.5, 125.0, and 250 μM (Lanes 1–9). Lane 10: minus enzyme control. Reaction products were separated in a 20% polyacrylamide gel containing 7 M urea. ^32^P -labeled phosphates are marked with an asterisk. Raw image of the gel is shown in Supplementary Fig. 2. The x- and y-axes are plotted on a linear scale. Points represent two independent assays (black and open circles). Raw data for Fig. 2 are included in Supplementary Data file. The continuous line represents the best fit for the Morrison equation^46^.

Since Tipiracil is an uracil derivative and Nsp15 is uridine-specific, we have speculated that the compound may inhibit the enzyme. This hypothesis was tested by using octamer RNA with single uridine site 5′AGGAAGU^32^pC, which under experimental conditions remains single-stranded and does not form duplexes. Upon endonuclease reaction, 3′-^32^P-labeled heptamer and unlabeled 3’CMP are produced. The transfer of the ^32^P from the pCp to the 3′ terminus of the heptamer is consistent with transphosphorylation mechanism. In the presence of 5 mM MnCl_2_, only partial cleavage is observed. This reaction is decreased by 50% in the presence of 7.5 μM Tipiracil (Fig. 2). At the higher Mn^2+^ ion concentration, the cleavage reaction proceeds to completion but no inhibition by Tipiracil can be measured.

**Fig. 3:**
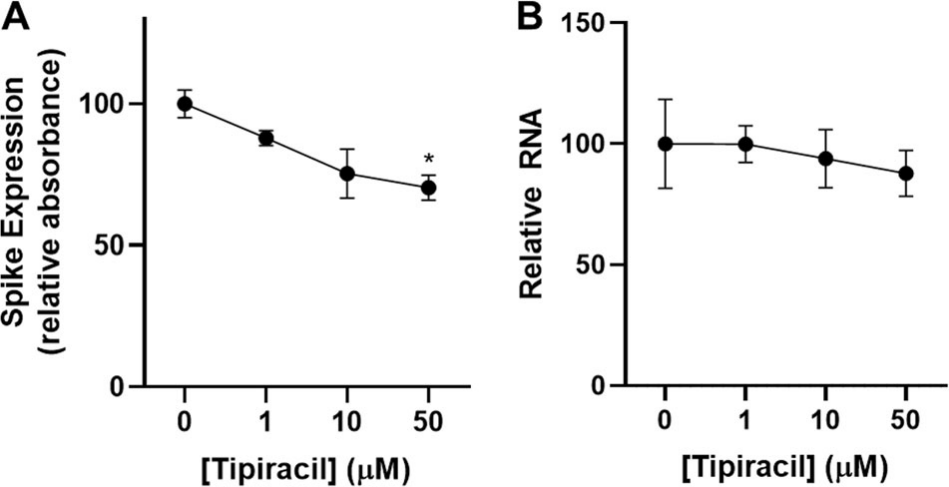
Inhibition of SARS-CoV-2 coronavirus by Tipiracil in whole-cell assays. A549-hACE2 cells were pre-treated with Tipiracil or carrier (0 mM) for 2 h and infected with CoV-2 at MOI 1. After 48 h, cells were harvested to check (**A**) spike protein and (**B**) RNA expression, *P < 0.05. Raw data for Fig. 3 are included in Supplementary Data file.

We have also performed S antigen ELISA assay in A549 cells and CoV-2 virus replication assay using qRT-PCR. Tipiracil was not affecting viability of cells but the inhibition of virus was found to be modest in the concentration range 1–50 μM (Fig. 3). This preliminary data suggests that the affinity of the compound may need to be improved in order to serve as an antiviral drug. Structural information is essential for this process (see below).

#### Structure determination and binding of ligands

**Fig. 4:**
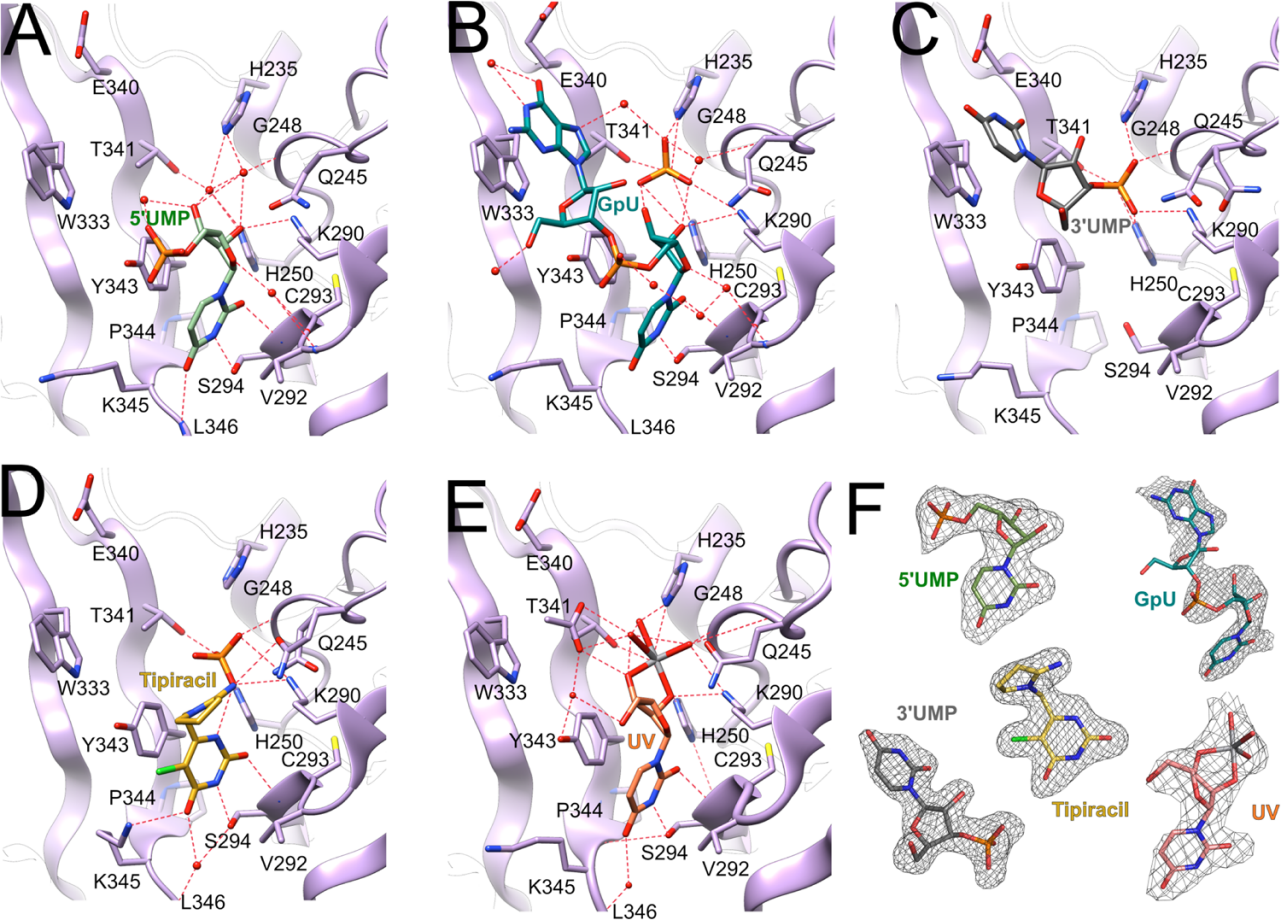
The structures of Nsp15 endoribonuclase with bound ligands. **A** 5′UMP in light green, **B** 5′GpU in teal, **C** 3′UMP in gray, **D** Tipiracil in yellow, and **E** uridine 2′,3′-vanadate in light coral. Phosphate ions are in orange/red. **F** Ligands electron density omit maps contoured at 3.2 σ except for the map for GpU which was contoured at 2.5 σ.

SARS-CoV-2 Nsp15 protein was crystallized with 5′UMP, 3′UMP, 5′GpU, uridine 2′,3′-vanadate (UV), and Tipiracil using methods described previously^8^ and the structures were determined at 1.82 Å, 1.85 Å, 1.97 Å, 2.25 Å, and 1.85 Å resolution, respectively. With exception of the complex with UV, crystals with ligands diffract to a higher resolution than the apoprotein (2.20 Å). Of note, we were unable to obtain co-crystals of Nsp15 with 5′AMP, 3′,5′-cyclic AMP, 5′GMP, 5′TMP, and 5′CMP using the same set of conditions as tested for 5′UMP. All structures were solved by molecular replacement using Nsp15 (PDB id: 6WVV) and refined as explained in Materials and Methods and Table 1. The majority of the uncleaved His-tag residues are not ordered and hence not visible but for all residues from Met1 to Gln347 and for all bound ligands the electron density is excellent (Fig. 4). In all five structures, ligands bind to the C-terminal catalytic domain active site (Fig. 4), though the exact positions vary, as described below. In 5′GpU and Tipiracil complexes, there is also a phosphate ion bound in the catalytic pocket. The compound binding is facilitated by side chains of seven conserved residues His235, His250, Lys290, Trp333, Thr341, Tyr343, and Ser294 and the main chain of Gly248, Lys345, and Val292, as well as water molecules (Fig. 4). One non-conserved residue, Gln245, is also involved through a water-mediated interaction. The interactions do not trigger any major protein conformational changes either globally or locally. In fact, the catalytic residues (His235, His250) and other active site residues discussed below maintain very similar conformations in all complexes (RMSD 0. 29 Å) over Cα atoms for residues His235, Gly248, His250, Lys290, Trp333, Thr341, Tyr343, and Ser294 in the pairwise superposition of complexes with the apo-structure (the highest is 0.39 Å for B chain of the Tipiracil complex and the lowest is 0.29 Å for the chain B of the 5′UMP complex). The position of phosphate ion or 3′-phosphoryl group of 3′UMP is also preserved. The vanadate moiety of UV overlaps with the bound phosphate. Specific interactions of ligands with the protein are described below.

**Table 1:** Data processing and refinement statistics.

Structure	Nsp15/5′UMP	Nsp15/3′UMP	Nsp15/5′GpU	Nsp15/Tipiracil	Nsp15/UV
Data processing					
Space group	P6_3_	P6_3_	P6_3_	P6_3_	P6_3_
Cell dimensions					
* a* = *b, c* (Å)	150.96, 111.30	150.94, 111.86	150.88, 111.79	150.86, 111.70	150.83, 110.73
* α, β, γ* (°)	90, 90, 120	90, 90, 120	90, 90, 120	90, 90, 120	90, 90, 120
Resolution range (Å)^a^	1.82 (1.82–1.85)	1.85 (1.85–1.88)	1.97 (1.97–2.00)	1.85 (1.85–1.88)	2.25 (2.25–2.29)
Unique reflections^a^	130,237 (6,453)	122,922 (6,120)	102,091 (5,100)	122,260 (5,907)	66,979 (3,067)
R-merge^*b*^	0.148 (1.879)	0.146 (1.587)	0.155 (1.452)	0.159 (1.350)	0.193 (1.308)
Mean I/sigma(I)	18.5 (1.0)	25.1 (1.5)	16.0 (1.30)	22.5 (1.10)	12.6 (1.0)
CC_1/2_^c^	0.985 (0.407)	0.999 (0.561)	0.972 (0.373)	0.987 (0.471)	0.985 (0.479)
Completeness (%)	100 (100)	100.0 (99.7)	100 (99.8)	99.8 (96.9)	98.6 (91.3)
Redundancy	7.5 (6.3)	12.8 (6.4)	6.2 (4.8)	11.2 (6.1)	7.0 (5.5)
Wilson B-factor (Å^2^)	38.46	17.1	37.60	30.97	42.0
Refinement					
Resolution range (Å)	1.82–40.63	1.85–45.19	1.97–49.39	1.85–42.45	2.25–49.37
Reflections work/test	123,356/6,406	108,782/5,676	96,700/5,021	115,822/6,000	63,079/3,363
R_work_/R_free_	0.170/0.195	0.166/0.189	0.157/0.185	0.171/0.194	0.167/0.192
Number atoms					
Protein	5,707	5,700	5,540	5,647	5,483
Ligand/ion	125	81	102	90	64
Water	486	718	432	702	265
Protein residues	348×2	348×2	348×2	348×2	348×2
RMSD (bonds) (Å)	0.006	0.002	0.002	0.015	0.002
RMSD (angles) (°)	0.762	0.534	0.405	1.251	0.494
Rotamer outliers (%)^d^	2.49	0.31	0.96	2.68	0.65
Clashscore	1.63	1.99	2.76	2.45	2.26
Average B-factor (Å^2^)	48.8	25.6	47.8	41.1	50.7
Protein	47.9	24.2	46.9	40.1	50.6
Ligand/ion	71.5	41.3	80.5	49.5	59.7
Water	52.8	33.7	51.0	48.5	50.3
Number of TLS groups	10	7	13	10	9
PDB ID	6WLC	6X4I	6X1B	6WXC	7K1L

^a^Values in parentheses correspond to the highest resolution shell.

The resolution was cut based on CC_1/2_ at higher than 0.35.

^b^Rmerge = ΣhΣj|Ihj–<Ih > |/ΣhΣjIhj, where Ihj is the intensity of observation j of reflection h.

^c^As defined by Karplus and Diederichs^47^.

^d^As defined by Molprobity^48^.

#### 5′UMP Binding

The model of uracil binding by Nsp15 was proposed based on the EndoU and RNase A structures^2^, speculating that Ser294 might be responsible for base discrimination. Here we describe experimental details of pyrimidine recognition. The base of 5′UMP forms van der Waals contacts with Tyr343 and several hydrogen bonds with active site residues, including sidechain OG and main-chain nitrogen atom of Ser294 (Fig. 4A). These interactions define O2 and N3 specificity. O4 interacts with the main chain nitrogen atom of Leu346 defining the uracil specificity. Potentially, cytosine and thymine pyrimidines may also bind—an amino group in position 4 of C should be compatible with the recognition pattern and a methyl group in position 5 should not interfere with binding either as uracil position 5 is solvent accessible. In fact, recognition of C has been reported for distantly related, though with similar active site, bacterial EndoU anticodon tRNase^14^. The ribose ring makes several hydrogen bonds with protein residues: (i) 2′OH interacts with Lys290 (NZ) and with NE2 of the catalytic His250, (ii) 3′OH makes water-mediated hydrogen bonds with catalytic His235 (NE2), His250 (NE2), Thr341 (OG1) and Gly248 (main chain nitrogen atom), and (iii) O4′ is hydrogen-bonded to main chain of Val292 via a water molecule. Interestingly, the 5′-phosphoryl group projects into solvent with no interaction with protein atoms. Its only ordered interaction is with 3′OH through a water molecule. This 5′-phosphoryl group location overlaps with that of Nsp15/5′GpU complex (see below). Also worth mentioning is that the ribose in the Nsp15/5′UMP together with the phosphate ion in the Nsp15/Tipiracil mimic 2′3′-cyclic phosphodiester. The structure with 5′UMP shows how the enzyme discriminates between uracil and purine bases with Ser294 serving as the key discriminatory residue, as has been hypothesized before^6,12,15,16^.

#### 5′GpU Binding

In RNA sequence containing uracil, such as 5′NpGpUpN3′, where N corresponds to any base, the Nsp15 cleavage would produce 5′NpGpU3′p, if transphosphorylation is followed by hydrolysis of 5′NpGpU2′3′p. In the crystal structure of Nsp15/5′GpU, the dinucleoside monophosphate binds to the active site with uracil interacting with Tyr343 and Ser294 (Fig. 4B), as seen in the Nsp15/5′UMP complex. However, the distance between O4 and the main chain nitrogen of Leu346 is too long to make a hydrogen bond. This implicates some flexibility at the protein C-terminus and suggests that the amino group in position 4 of cytosine can be accommodated, as we suggested above and reported previously^8^. The guanine ring is stacking against Trp333 and makes two hydrogen bonds with water molecules. The absence of defined base-side chain interactions suggests a lack of specificity for this site in the substrate sequence. The Nsp15/5′GpU complex binds also a phosphate ion in the active site, most likely from the crystallization buffer. The ion interacts with the protein side chains (His235, His250, Thr341, and Lys290) and uridine ribose 2′OH and 3′OH groups. It most likely mimics the binding of scissile phosphoryl group of the substrate. The backbone phosphoryl group (5′ of U) faces solvent as in the Nsp15/5′UMP complex and makes a hydrogen bond to a water molecule. This structure and the Nsp15/5′UMP complex illustrate location and specificity determinants of the uridine with a 5′-phosphoryl group. The binding of guanine in the structure identifies a strong base binding site at Trp333, which, however, may not necessarily be dedicated to 5′end of the oligonucleotide (see discussion below).

#### 3′UMP Binding

We co-crystallized Nsp15 with 3′UMP, assuming that the enzyme would dock it in a manner expected for uridine monophosphate nucleotide in the contexts of larger RNA substrate, preserving the uracil specific interactions described above. Surprisingly, the uracil base is anchored by Trp333 in the guanine site observed in the Nsp15/5′GpU complex (Fig. 4C), confirming that this site can accommodate purine and pyrimidine bases. The 3′-phosphoryl group occupies the phosphate ion site created by His235, His250, and Thr341, as observed in Nsp15/5′GpU. It is surprising that uracil does not go into its dedicated site, but the result demonstrates higher affinity for the base in the Trp333 site than in uracil-recognition site, potentially governed by the strong stacking interactions with the aromatic side chain that take precedence over hydrogen bonds observed in the uracil binding of 5′UMP. The identity of the Trp333-interacting base is irrelevant, especially given that the enzyme’s substrate is most likely a larger RNA molecule.

#### Tipiracil binding – a uracil derivative and Nsp15 inhibitor

**Fig. 5:**
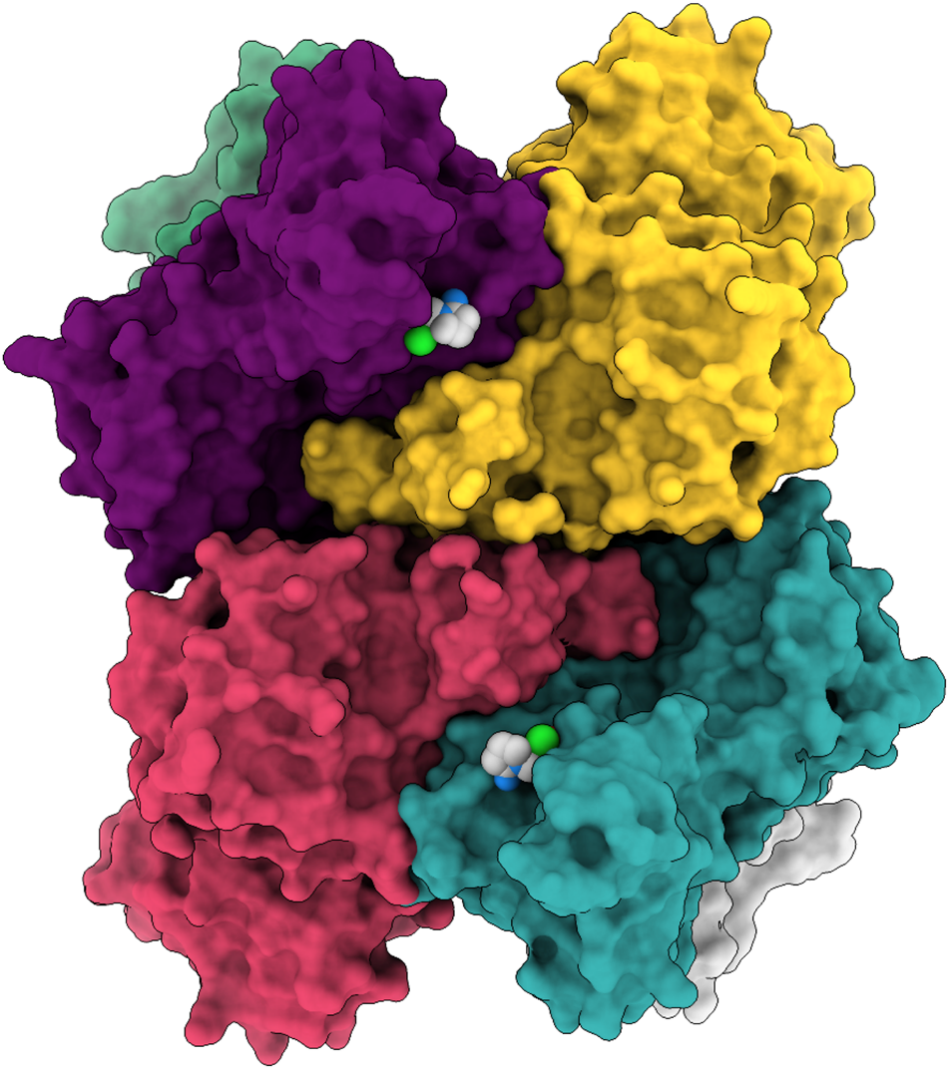
Structure of SARS-CoV-2 hexamer with the bound Tipiracil in surface representation. Tipiracil bound to each subunit active site is shown with all atoms in color spheres (carbon, chlorine, nitrogen, oxygen in white, green, blue, and red, respectively).

The Tipiracil molecule binds to the uracil site as observed in 5′UMP and 5′GpU (Figs. 4D and 5). The molecule makes several substrate analog-like interactions. The uracil ring stacks against Tyr341 and makes hydrogen bonds with Ser294 (interacting with O2 and N3), Lys345 (O4), and His250. The carbonyl group of Leu346 makes a water-mediated hydrogen bond in the structure with Tipiracil (Fig. 4D). The N1 atom makes a hydrogen bond with a phosphate ion and through it connects to Lys290. There are also two water-mediated interactions to Ser294 and the main-chain carbonyl oxygen atom of Val292. Iminopyrrolidin nitrogen atom binds to Gln245, representing the only interaction unique to the ligand. Nsp15 binds Tipiracil in its active site in a manner compatible with competitive inhibition and the compound and its derivatives may serve as inhibitors of the enzyme. This structure suggests that uracil alone may have similar inhibitory properties and provides basis for the uracil scaffold-based drug development.

#### Binding of uridine 2′,3′-vanadate – S_N_2 transition state analog

**Fig. 6:**
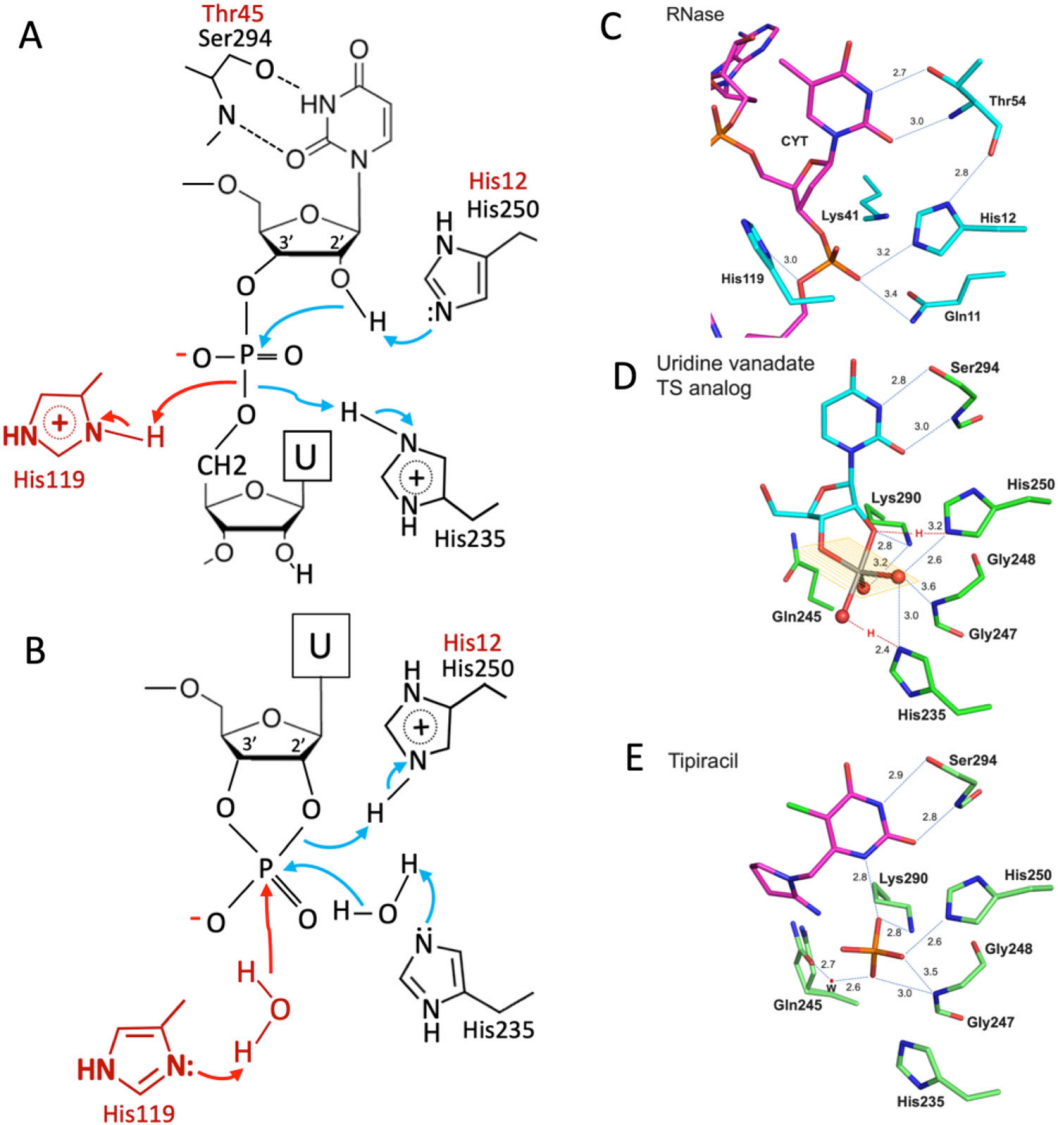
The reaction mechanism and comparison of Nsp15 and RNase A active sites. **A**, **B** Schematic drawings of reaction mechanism of Nsp15. **A** Transphosphorylation reaction that generates 2′,3′-cyclic phosphodiester. **B** Hydrolysis reaction from transition state. Residues shown in red are for RNase A and in black for Nsp15. Arrows depict electron transfer. The active site of RNase A (**C**) is compared with that of Nsp15 in the complex with transition state analog uridine 2′,3′-vanadate (**D**), and with Tipiracil (**E**). The transition state of the phosphate is present in both reactions.

The structure of the complex with UV was determined at 2.25 Å resolution. UV binds to the uridine binding site as observed in the structures with 5′UMP, GpU, and Tipiracil. The uracil moiety is recognized by Ser294 and Leu346 defining the uracil recognition and interacts also with Tyr343 (Fig. 4E). The pentavalent vanadate is covalently bound to 2′ and 3′ ribose oxygen atoms and it corresponds to the S_N_2 transition state^17–21^. The metal ion, O3′, and two vanadate oxygen atoms are in a plane. One oxygen (O2′) atom below the plane is interacting with His250 and the other oxygen above the plane is in contact with His235 (Figs. 4E and 6B). The vanadate position is consistent with 3′-phosphoryl group that is cleaved in the RNA substrate and observed in the GpU and Tipiracil complexes. The vanadate interacts with key catalytic site residues including His250, His235, Lys290, Thr341, Gly247 and has several water-mediated interactions (Fig. 4E). Particularly, Lys290 makes hydrogen bonds to O2′ and one of the vanadate oxygen atoms in the plane reminiscent of Lys41 in RNase A. These interactions confirm previous experimental observations for Nsp15 and are consistent with the proposed S_N_2 mechanism. The structure also provides insights into the structure of 2′,3′-cyclic phosphodiester, which is the product of transphosphorylation reaction. This structure suggests that Nsp15 should follow a two-step reaction mechanism with the final product being 3′UMP.

#### Compounds influence on Nsp15 thermal stability

The differential scanning fluorimetry experiments (DSF) showed that melting temperature (Tm) of the Nsp15 in a presence of Tipiracil, 5′GpU, 5′UMP, 3′UMP, and 3′TMP is approximately 60.5 °C under-investigated conditions with buffer that contains 10 mM MnCl_2_ (Supplementary Fig 1. A, B). Depicted local minima of the Nsp15 first derivative of fluorescence signals have the same Tm values as a control. The denaturation profile of the Nsp15 in the presence of Tipiracil is broader and consistently shifted (0.5 °C) to higher temperatures (Supplementary Fig 1. A). Therefore, DSF results indicate the small increase of stability of the Nsp15/ Tipiracil complex in comparison to the control sample and other tested Nsp15 complexes with 5′GpU, 5′UMP, 3′UMP, and TMP. Additional change in the Nsp15 Tm is observed at 83 °C (Supplementary Fig. 1B). This is caused by all ligands and may be related to increased stability of the hexamer or EndoU catalytic domain. In the presence of Mn^2+^ ions the main Tm of Nsp15 is increased from 56.5 °C to 60.5 °C and is Mn^2+^ concentration-dependent. Interestingly, at 5 mM and 20 mM concentrations of Mn^2+^ a new local Tm minimum is observed at 83 °C potentially suggesting increased stability of the EndoU domain (Supplementary Fig. 1C).

#### Comparison of CoV-2 Nsp15 active site with RNase A – mechanistic implications

**Fig. 7:**
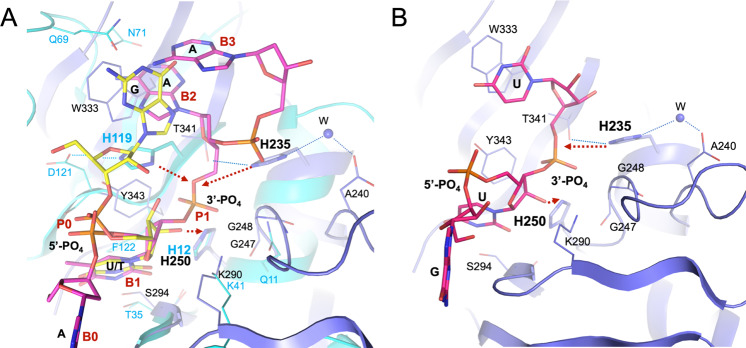
Comparison of Nsp15 and RNase A active sites. **A** Nsp15/5’GpU complex compared with RNase A/5’dApdTpdApdA complex (PDB id 1RCN). The least square superposition is calculated with the Nsp15/RNase A residues H250/H12, S294/T35, K290/K41, and U/T bases using COOT (RMSD 0.6 Å on Cα atoms). Nsp15 is shown in dark blue, RNase A in aqua, 5’GpU in yellow and 5’dApdTpdApdA oligonucleotide in pink. Base positions B0-B3 and phosphoryl groups positions P0 and P1 are indicated in red (bold). **B** Model of 5'GpUpU oligonucleotide binding to the Nsp15 active site. Red arrows show proton flows (extraction and donation) in **A** & **B**.

Our structures of complexes with nucleotides can inform catalytic mechanism of Nsp15 endoribonuclease. We compared our structures with eukaryotic RNase A, a very well-studied model system^22^ (Fig. 7). RNase A recognizes pyrimidine nucleotides in RNA, preferring C over U, and catalyzes a two-step reaction, the transphosphorylation of RNA to form a 2′,3′-cyclic CMP intermediate followed by its hydrolysis to 3′CMP. Nsp15 also recognizes pyrimidines, preferring U over C, and was proposed to catalyze an analogous reaction^9^. In RNase A the base selectivity is achieved by Thr45 that forms specific hydrogen bonds similar to those created by Ser294 in the Nsp15/5′UMP, Nsp15/Tipiracil, and Nsp15/5′GpU complexes (Fig. 7). In RNase A the transphosphorylation reaction proceeds via an asynchronous concerted general acid/base mechanism involving His12, His119, and Lys41^23^. In this mechanism the 2′OH proton is transferred to the deprotonated form of His12 to activate the 2′O nucleophile. Then, the protonated His119 donates a proton to the departing 5′OH group. Lys41 function is to stabilize the negative charge that accumulates on the nonbridging phosphoryl oxygen atoms in the transition state. In the hydrolysis step the role of two histidine residues are inverted. The Nsp15 mechanism of catalysis is consistent with that of RNase A as observed in the structure of the complex with UV transition state (Figs. 4E and 7B). The 2′OH proton is transferred to the deprotonated form of His250 to activate the 2′O nucleophile, the protonated His235 donates a proton to the departing 5′OH group and Lys290 stabilizes the negative charge of the vanadate ion.

The RNase A active site is well organized and has several distinct pockets for binding RNA substrates (e.g., bases B1, B2, B3, and phosphoryl groups P0, P1, and P2) (for review see^24,25^). The B1 site provides base specificity and P1 site binds the scissile phosphoester bond. B0 represent the binding site for the base upstream of the B1-P1 cleavage site. In the RNase A complex with the 5′dApdTp↓dApdA deoxyoligonucleotide (PDB id: 1RCN,^26^), B0 interacts with adenine, B1 binds thymine, etc. The P1 site represents the above-mentioned catalytic machinery consisting of His12, His119, and Lys41, and Lys41 assisted by Gln11 in phosphoryl binding.

Structural alignment of the Nsp15 and RNase A catalytic site residues and RNA ligands shows that they adopt similar positions in the two enzymes, despite sequence and structure dissimilarity. Specifically, His250, Ser294, and Lys290 virtually overlap with the His12, Thr45, and Lys41. His250, by analogy His12 in RNase A, is in position to serve as the key residue in deprotonation of 2′OH. It is close to 2′OH in both 5′GpU and 5′UMP structures (3.2 and 3.8 Å respectively) and is in similar orientation in the complex of RNase A with the DNA substrate analog (Fig. 6). Therefore, His250 is very likely to directly activate 2′OH. Lys290 seems to play a role of Lys41 in RNase A. Main chain amide of Gly248 may provide function of Gln11 in binding to the substrate phosphoryl group. If His250 is a base in Nsp15 then the His235 must be a proton donor for the departing 5′OH group and equivalent of His119 of RNase A. However, these residues are ~8 Å apart and they approach the phosphoryl group from different directions (Fig. 6A). In RNase A, His119 forms a hydrogen bond with Asp121 which may provide proton for the reaction. The structural environment for His235 is different in Nsp15. Thr341 forms a hydrogen bond with His235 and there is also Asp240 further away that makes a water-mediated hydrogen bond with His235. In the hydrolysis step of converting the 2′3′-cyclic phosphate back to 2′OH and 3′-phosphoryl group the roles of histidine residues are reversed and now His235 must be a base deprotonating a water molecule and His250 serves as a proton donor for the 2′OH leaving group. A different set of interactions involving Nsp15 and RNase A catalytic residues may explain why Nsp15 activity is more sensitive to low pH and it is expected that kinetics of the reactions will be different.

Besides similarities in P1, the two enzymes share the organization of B1 pocket. Here, Nsp15 has a very well-defined uracil recognition site made of Ser294, Tyr343, and Leu346 that are equivalent to Thr45, Phe120, and Ser123 in RNase A. Further extrapolation from the RNase A model of the deoxyoligonucleotide binding allows us to hypothesize that B2 site, dedicated to the base on the 3′ end of scissile bond, in Nsp15 is created by Trp333. Its sidechain provides stacking option for a base with no base selectivity function. Yet in our Nsp15/5′GpU structure this site is occupied by the G base located on the 5′end of U. We speculate, that for oligonucleotides that are flanked on both sides of U, mimicking the RNase A ligand, 5′ guanine (or a different base) may adopt position of 5′dA that locates to the B0 site owing to the rotation of the P-5′O bond. Then, the available Trp333 base binding site can accept base on the 3′ end of the uridine moiety, somewhat resembling our Nsp15/3′UMP structure. When we combine oligonucleotides from our structures with RNase A/DNA complex a plausible model can be built of 5’GpUp↓U nucleotide bound to the Nsp15 active site (Fig. 6B). This model underscores importance of conserved Trp333 in anchoring RNA in the active site. While Ser294 is the key residue in discriminating base, the hydrophobic interaction with Trp333 may be a significant force for ligand docking. The B3 site is not easily identifiable in the available Nsp15 structures. In addition, unlike in RNase A, where all P0-P2 subpockets contribute to the backbone binding, in Nsp15 only P1 site is currently well-defined. 5′-phosphoryl groups in position P0 of the ligands do not form any direct contacts with the protein, while RNase A P0 site has Lys66 participating in RNA binding. P2 site of RNase A is created by Lys7 and it appears that His243 may fulfill such a role in Nsp15.

## Conclusions

The active site of Nsp15 can accommodate all five ligands, three nucleotides (5′UMP, 3′UMP, 5′GpU), one synthetic analog (Tipiracil), and uridine 2′,3′-vanadate—transition state analog. Interactions with these small molecules seem to stabilize the active site residues and entire catalytic EndoU domain resulting in a better-ordered protein. Previous mutational studies demonstrated that two histidine (His235 and His250) and one serine (Ser294) residues are essential for SARS-CoV Nsp15 activity. We, for the first time, illustrated how Ser294 participates in uracil (or pyrimidine) recognition and we concur with previous suggestions that His250 may be key to deprotonate the 2′OH to allow nucleophilic attack on the phosphoryl group. Comparisons of Nsp15 with RNase A active site display some similarities (active site residues conservation) and indicate a common catalytic mechanism with a two-step reaction releasing 3′UMP, but organization of the RNA binding site is distinct, especially at sites more distant from the elements crucial for chemistry.

Our structures are consistent with the binding of single-stranded nucleic acids, such as loops or bulges, as was shown for Nsp15s and other EndoUs. Both uracil and preceding base must be in an unpaired region in order to bind to Nsp15, which is consistent with degradation of polyU tracks as reported recently^5^. Our structures show that the preceding base can be guanine or uracil, or other bases as well (see below). Accommodation of 5′GpU and 5′GpUpU is in agreement with previous reports of guanine preference in anterior position with respect to U and efficient degradation of polyU tracks. However, SARS-CoV-2 Nsp15 can cleave RNA, at high Mn^2+^ concentrations, at uridine sites connected to any base in anterior position; for example, the synthetic EndoU substrate used for the nuclease assay has A.

Nsp15 binds nucleotides to the catalytic domain of each monomer independently, therefore it is not clear why the hexamer is required for the EndoU activity of the enzyme. We show that Nsp15 can bind and hydrolyze 4, 7, and 20 nucleotide long RNA. It is possible that the hexamer is needed to bind longer RNA substrates or is involved in interactions with other proteins (for example Nsp8 and Nsp12) within RTC^27^.

The role for metal ion requirement remains a puzzle. Although the Mn^2+^ dependence has been reported for some EndoU members and appears to be a common feature of NendoU subfamily, the metal-binding site was never located. Past studies indicated that single strand polyU RNA is relatively unstructured under most conditions. It was showed that Nsp15 cleavage is RNA sequence and structure dependent^6^. It is possible that the metal is required for maintaining conformation of the RNA substrate during catalysis. For example, metal ions like Mg^2+^, Mn^2+^ and Zn^2+^ form complexes with purine nucleotides to affect outcome of many enzymatic reactions^28^. Binding of Mn^2+^ ions to RNA molecules may dramatically transform their structure, as it was shown for riboswitch^29^ and the *Bacillus subtilis* M-box aptamer that sequence contains U track (PDB id: 3PDR,^30^). As manganese can exist in several oxidation states, it is also possible that metal redox properties can affect protein and RNA interactions and chemistry.

Tipiracil, an uracil derivative, binds to Nsp15 uracil site in a manner consistent with competitive inhibition. In vitro it inhibits Nsp15 RNA nuclease activity and shows modest inhibition of CoV-2 virus replication in the whole cell assay. While the compound itself is not optimal for the therapeutic applications, our work shows that uracil and its derivatives may represent a plausible starting point for nucleotide-like drug development. Moreover, interaction of Trp333 with bases may provide additional site to build much higher affinity inhibitors.

## Materials and methods

3′-CMP was obtained from Sigma. Crude [gamma-^32^P]ATP was purchased from PerkinElmer. T4 Polynucleotide Kinase (3’ phosphatase minus) was from New England BioLabs. All other chemicals were reagent grade.

### Cytidine and uridine 3,5′-bisphophates synthesis

[5′-^32^P]pCp was prepared by phosphorylation of 3′-CMP as described by England et al.^31^.

### RNA synthesis

RNA eicosamers were synthetized by runoff transcription of synthetic double-stranded DNA templates published by Sherlin et al.^32^. 5′-^32^P-labeled RNA eicosamers and 3′-^32^P-labeled RNA octamers were prepared according to Zwieb et al.^33^ and England et al.^31^, respectively.

### Protein purification

Protein was expressed and purified using protocol developed by Kim et al.^8^. Briefly, a 4 l culture of LB Lennox medium was grown at 37 °C (190 rpm) in presence of ampicillin 150 mg/ml. Once the culture reached OD_600_ ~1.0, the temperature setting was changed to 4 °C. When bacterial suspension cooled down to 18 °C it was supplemented with the following components to indicated concentration: 0.2 mM IPTG, 0.1% glucose, 40 mM K_2_HPO_4_. The temperature was set to 18 °C for 20 h incubation. Bacterial cells were harvested by centrifugation at 7,000 *g* and cell pellets were resuspended in a 12.5 ml lysis buffer (500 mM NaCl, 5% (v/v) glycerol, 50 mM HEPES pH 8.0, 20 mM imidazole, and 1 mM TCEP) per liter culture and sonicated at 120 W for 5 min (4 s ON, 20 s OFF). The cellular debris was removed by centrifugation at 30,000 *g* for 1 h at 4 °C. Supernatant was mixed with 4 ml of Ni^2+^ Sepharose (GE Healthcare Life Sciences) equilibrated with lysis buffer supplemented to 50 mM imidazole pH 8.0 and suspension was applied on Flex-Column (420400-2510) connected to Vac-Man vacuum manifold (Promega). Unbound proteins were washed out via controlled suction with 160 ml of lysis buffer (50 mM imidazole). Bound proteins were eluted with 20 ml of lysis buffer supplemented imidazole to 500 mM pH 8.0. Then 1 mM TCEP was added followed by Tobacco Etch Virus (TEV) protease treatment at 1:20 protease:protein ratio. The solution was left at 4 °C overnight. For this particular construct, TEV protease was not able to cleave off the His tag. Nsp15 was successfully separated from TEV protease on Superdex 200 column equilibrated in lysis buffer where 10 mM β-mercaptoethanol was replaced by 1 mM TCEP. Fractions containing Nsp15 were collected. Lysis buffer was replaced on 30 kDa MWCO filter (Amicon-Millipore) via 10× concentration/dilution repeated 3 times to crystallization buffer (150 mM NaCl, 20 mM HEPES pH 7.5, 1 mM TCEP). Final concentration of Nsp15 was 19 mg/ml.

### Crystallization of complexes

Crystallization experiments were conducted using previously published protocols^8^. Except for Nsp15/UV, all complexes Nsp15/5′UMP, Nsp15/3′UMP, Nsp15/5′GpU, and Nsp15/Tipiracil were prepared by mixing 5–15 mM of each ligand with 0.2 mM Nsp15 and incubate for at least 30 min before crystallization. Crystals of Nsp15/3′UMP, Nsp15/5′GpU, and Nsp15/Tipiracil, the hexagonal crystal form, in *P*6_3_ space group, were obtained from MCSG1 screen A3 condition containing 0.2 M sodium chloride, 0.1 M sodium/potassium phosphate pH 6.2, 10 %(w/v) PEG8000. These crystals diffracted x-rays to 1.85, 1.93, and 1.85 Å for Nsp15/3′UMP, Nsp15/5′GpU, and Nsp15/Tipiracil, respectively. The crystals of Nsp15/5’UMP and Nsp15/uridine vanadate complexes grew from the Crystal Screen Classic HTP (Jena Bioscience) C2 condition containing 16 %(w/v) PEG4000, 0.1 M Tris pH 8.5, 0.2 M sodium acetate and diffracted to 1.82 Å and 2.25 Å respectively. To achieve higher occupancies of the ligands in the structures, except for uridine vanadate, each ligand compound was added to the cryo-solution and the co-crystal was soaked for 2–3 min before frozen in the liquid nitrogen.

### Endoribonuclease assays

Typical reaction contained 5 × 10^5^ CPM of 5′-^32^P-labeled RNA eicosamers or 3′-^32^P-labeled RNA octamers (0.5 µM final RNA concentration) and 10 nM SARS-CoV-2 Nsp15 in 20 mM HEPES-KOH (pH 7.5), 50 mM KCl, 1 mM DTT and either 5 mM or 11 mM MnCl_2_. To inhibit endonuclease, Tipiracil dissolved in water was added. Following incubation at 30 °C for up to 60 min, the reactions were stopped by adding an equal volume of a gel-loading buffer containing 95% formamide, 10 mM EDTA, and 0.025% SDS. The products were analyzed in 20% polyacrylamide gels (acrylamide/bisacrylamide ratio, 19:1) buffered with 0.5x Tris-borate-EDTA containing 7 M urea.

### Synthesis of uridine 2′,3′-vanadate

The protocol was modified from the references^34,35^ to yield higher concentration of uridine vanadate. 1 mg of ammonium meta vanadate is dissolved in 200 μl of 60 °C water and 2.93 mg of uridine is added then incubated for 10 min at the same temperature to obtain ~ 40 mM uridine vanadate solution. Without further purification, this solution and protein were mixed in a 10:1 molar ratio to set up crystals.

### Cells and virus

A549 cells expressing human ACE2 (kind gift of Dr. Benjamin R. tenOever, Mt. Sinai School of Medicine) were infected under biosafety level 3 conditions with SARS-CoV-2 (nCoV/Washington/1/2020, kindly provided by the National Biocontainment Laboratory, Galveston, TX).

### Cell viability

Cell viability was measured by staining with CellTracker™ Red CMTPX (ThermoFisher Scientific). SARS-CoV-2 infected cell was stained with 2 µM of CellTracker™ Red CMTPX for 30 min and then detected by Tecan Infinite m200 (Tecan) at ex577/em602 nm. After reading, cells were fixed by 10% neutral buffered formalin (NBF) for immunohistochemistry assay.

### Immunostaining against Spike protein

Immunostaining was performed on 10% NBF fixed SARS-CoV-2 infected cells in 96-well plate. After fixation, 10% NBF was removed, and cells were washed with PBS, followed by washing with PBS-T (0.1% Tween 20 in PBS), and then blocked for 30 min with PBS containing 1% BSA at room temperature. After blocking, endogenous peroxidases were quenched by 3% hydrogen peroxide for 5 min. Then, cells were washed with PBS and PBS-T and incubated with a monoclonal mouse-anti- SARS-CoV-2 spike antibody (GeneTex, 1:1000) in PBS containing 1% BSA overnight at 4 °C. Primary antibody was washed with PBS and PBS-T and then cells were incubated in secondary antibody (ImmPRESS Horse Anti-Mouse IgG Polymer Reagent, Peroxidase; Vector Laboratories) for 60 min at room temperature. After washing with PBS for 10 min, color development was achieved by applying diaminobenzidine tetrahydrochloride (DAB) solution (Metal Enhanced DAB Substrate Kit; ThermoFisher Scientific) for 30 min and detected by Tecan Infinite m200 (Tecan) at 492 nm after replacing DAB solution to PBS. The result was normalized by cell viability.

### RNA Extraction and qRT-PCR

Total RNA from SARS-CoV-2 infected cells was isolated using a NucleoSpin 96 RNA kit following the manufacturer’s instructions (Macherey-Nagel). SARS-CoV-2 RNA was quantified by qRT-PCR using SuperScript™ III Platinum™ One-Step qRT-PCR Kit w/ROX (ThermoFisher Scientific) and normalized using Eukaryotic 18 S rRNA Endogenous Control (VIC™/MGB probe, Applied Biosystems) via a StepOnePlus real-time PCR system (Applied Biosystems). All reactions performed in a dual-plex qRT-PCR using the CDC-recommended primers for N1. Primer and probe sequences are as follows: 2019-nCoV_N1 Forward Primer (2019-nCoV_N1-F), GACCCCAAAATCAGCGAAAT; 2019-nCoV_N1 Reverse Primer (2019-nCoV_N1-R), TCTGGTTACTGCCAGTTGAATCTG; Probe (2019-nCoV_N1-P), FAM-ACCCCGCATTACGTTTGGTGGACC-BHQ1.

### Protein differential scanning fluorimetry

The Nsp15 differential scanning fluorimetry assays were done in a buffer 20 mM Tris pH 7.5, 100 mM NaCl, 1 mM TCEP that was supplemented with SYPRO Orange (Invitrogen) dye^36^ to 5x final concentration. We used 10 µM of the Nsp15 with addition of 1 mM of Tipiracil, 5′GpU, 5′UMP, 3′UMP, TMP, respectively to get the final molar ratio of protein to ligand 1:100. Additionally, the samples with ligands were supplemented with 10 mM MnCl_2_. After 30 min of incubation of samples at room-temperature fluorescence measurements were done using CFX Connect Real-Time System (BioRad). Fluorescent signal was detected with temperature rate 1 °C per 60 s. Graphs represents the first derivative of observed fluorescence signal. Samples were measured in triplicates and the representative standard deviation of measurements are depicted for Nsp15 control sample and complex of the Nsp15 with the addition of Tipiracil.

### Data collection, structure determination, and refinement

The x-ray diffraction experiments were carried out at the Structural Biology Center 19-ID beamline at the Advanced Photon Source, Argonne National Laboratory. The diffraction images were recorded at 100 K at the wavelength of 0.9793 Å from all crystal forms on the PILATUS3 X 6 M detector using 0.5° rotation and 0.5 s exposure for 140°, 180°, 110°, 210°, and 125° for Nsp15/5′UMP, Nsp15/3′UMP, Nsp15/5′GpU, Nsp15/Tipiracil, and Nsp15/UV, respectively. The data were integrated and scaled with the HKL3000 suite^37^. Intensities were converted to structure factor amplitudes in the Ctruncate program^38,39^ from the CCP4 package^40^ and using the apo-form SARS-CoV-2 Nsp15 structure (PDB id: 6VWW) as a search model, the structures were determined using MOLREP^41^, all implemented in the HKL3000 software package. The initial solutions were refined, both rigid-body refinement and regular restrained refinement by REFMAC^40,42^ as a part of HKL3000. The models including the ligands were manually adjusted using COOT^43^ and then iteratively refined using COOT and PHENIX^44^. Throughout the refinement, the same 5% of reflections were kept out throughout from the refinement (in both REFMAC and PHENIX refinement). The stereochemistry of the structure was checked with PROCHECK^45^ and the Ramachandran plot and validated with the PDB validation server. For the structures Nsp15/5′UMP, Nsp15/3′UMP, Nsp15/5′GpU, Nsp15/Tipiracil, Nsp15/UV, Ramachandran favored/allowed/outliers are 98.12/1.88/0.0, 97.38/2.47/0.15, 97.39/2.61/0.0, 97.97/2.03/0.0, and 96.96/3.04/0.0%, respectively. The data collection and processing statistics are given in Table 1.

### Statistics and reproducibility

The results included in this manuscript can be reproduced by following protocols and using materials described in Materials and Methods.

### Reporting summary

Further information on research design is available in the Nature Research Reporting Summary linked to this article.

## Supplementary information

Supplemental Information

Description of Additional Supplementary Files

Supplementary Data 1

Reporting Summary

## Data Availability

The structural datasets generated during the current study are available in the Protein Data Bank repository (https://www.rcsb.org/) under accession codes: 6WLC, 6X4I, 6X1B, 6WXC, and 7K1L. Diffraction images are available on the server in Dr. W. Minor laboratory https://proteindiffraction.org. Plasmid for expression Nsp15 (NR-52426 Vector pMCSG53 Containing the SARS-Related Coronavirus 2, Wuhan-Hu-1 Non-Structural Protein 15) is available in the NIH the BEI Resources Repository (https://www.niaid.nih.gov/research/bei-resources-repository). All other data generated during the current study including the raw kinetic and biophysical data are available upon request.
