# Supplementary material for: Tipiracil binds to uridine site and inhibits Nsp15 endoribonuclease NendoU from SARS-CoV-2
Source: Commun Biol. 2021 Feb 9;4:193. doi: 10.1038/s42003-021-01735-9 (PMC7873276; doi:10.1038/s42003-021-01735-9)
Supplement: Supplementary file 1 — Supplemental Information [file 42003_2021_1735_MOESM1_ESM.pdf]

SUPPLEMETARY MATERIALS

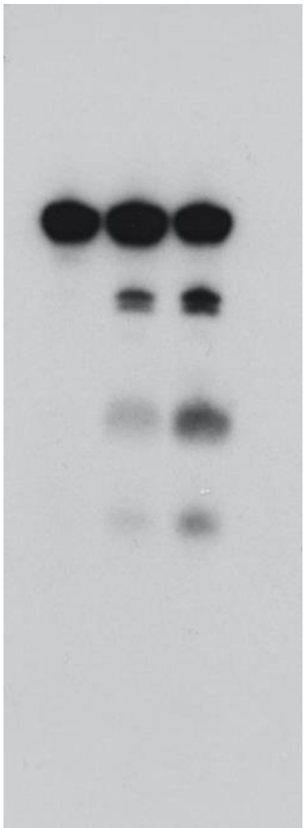

Supplementary Figure 1A. Uridine-specific endoribonuclease activity of SARS-CoV-2 Nsp15 in the presence of 5 mM Mn^{2+}. Raw autoradiogram of 20% polyacrylamide gel containing 7 M urea showing separation of the reaction products 5'-^{32}P-labeled RNA eicosamers incubated at 37°C with Nsp15.

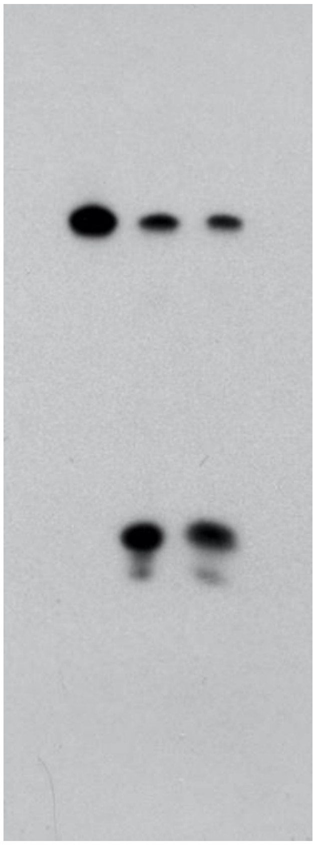

Supplementary Figure 1B. Uridine-specific endoribonuclease activity of SARS-CoV-2 Nsp15 in the presence of 11 mM Mn^{2+}. Raw autoradiogram of 20% polyacrylamide gel containing 7 M urea showing separation of the reaction products 5'-^{32}P-labeled RNA eicosamers incubated at 37°C with Nsp15.

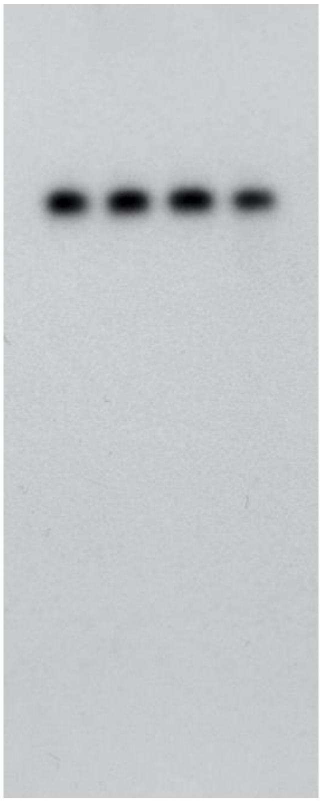

Supplementary Figure 1C. Uridine-specific endoribonuclease activity of SARS-CoV-2 Nsp15 on eicosamer that does not contain uridine in its sequence in the presence of 11 mM Mn^{2+}. Raw autoradiogram of 20% polyacrylamide gel containing 7 M urea showing separation of the reaction products 5’-^{32}P-labeled RNA eicosamers incubated at 37°C with Nsp15.

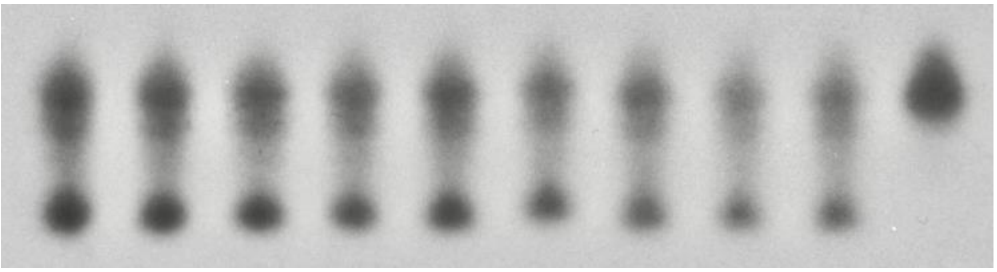

Supplementary Figure 2. Inhibition of SARS-CoV-2 Nsp15 endoribonuclease by Tipiracil. Raw autoradiogram shown as insert in Fig. 2 of a 20% polyacrylamide gel containing 7 M urea showing separation of RNA ^{32}P labeled heptamers (5’AGGAAGU^{32}p) from octamers 5’ AGGAAGU^{32}pCp. These oligonucleotides were incubated at 30°C with Nsp15 in the presence of increasing concentrations of Tipiracil.

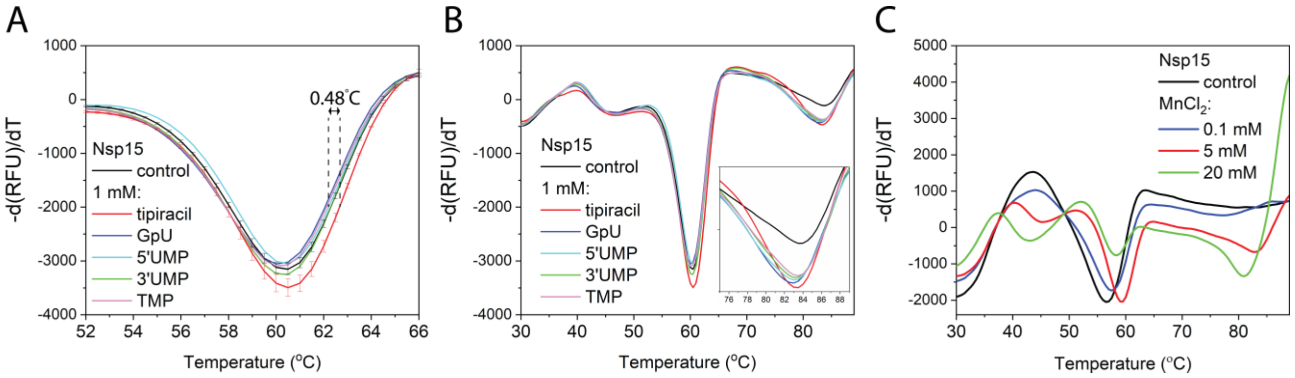

Supplementary Figure 3. Denaturation curves of the Nsp15 in a presence of Tipiracil, 5’GpU, 5'UMP, 3'UMP, TMP (A, B). Thermal stability of Nsp15 after addition of manganese ions (C). Nsp15 samples were labeled with SYPRO orange dye [46].
