# Supplementary material for: Tipiracil binds to uridine site and inhibits Nsp15 endoribonuclease NendoU from SARS-CoV-2
Source: Commun Biol. 2021 Feb 9;4:193. doi: 10.1038/s42003-021-01735-9 (PMC7873276; doi:10.1038/s42003-021-01735-9)
Supplement: Supplementary file 2 — Description of Additional Supplementary Files [file 42003_2021_1735_MOESM2_ESM.pdf]

Description of Additional Supplementary Files

File Name: Supplementary Data 1

Description: Raw data for Figures 2 and 3.
